# Validation and Implementation of a Highly Sensitive and Efficient Newborn Screening Assay for Mucopolysaccharidosis Type II

**DOI:** 10.3390/ijns6040079

**Published:** 2020-10-14

**Authors:** Heather Bilyeu, Jon Washburn, Lacey Vermette, Tracy Klug

**Affiliations:** 1 Missouri State Public Health Laboratory, 101 N. Chestnut Street, PO Box 570, Jefferson City, MO 65102-0570, USA; heather.bilyeu@health.mo.gov (H.B.); lacey.vermette@health.mo.gov (L.V.); 2 Baebies, Inc., PO Box 14403, Durham, NC 27709, USA; JWashburn@baebies.com

**Keywords:** Hunter syndrome, mucopolysaccharidosis II, newborn screening, analytical validation, clinical validation

## Abstract

Mucopolysaccharidosis Type II (MPS II), also known as Hunter syndrome, is a lysosomal storage disorder (LSD) caused by a deficiency of the lysosomal enzyme iduronate-2-sulfatase (IDS). MPS II satisfies all criteria defined by the Advisory Committee on Heritable Disorders in Newborns and Children (ACHDNC) for inclusion in the Recommended Uniform Screening Panel (RUSP) for newborn screening, apart from the fact that only minimal prospective population screening data are available. This report details the analytical validation, clinical validation, and implementation of a fluorometric assay for measurement of IDS activity in newborn dried blood spot (DBS) specimens at the Missouri State Public Health Laboratory (MSPHL). The assay is performed in a microwell plate format requiring approximately 15 min of hands-on time per plate and an incubation time of two hours. The analytical validation of this assay included linearity, analytical sensitivity, precision, and carry-over testing. Clinical validation was completed using more than 5000 deidentified presumptive normal newborn DBS specimens as well as seven specimens from patients known to be affected with MPS II. Following validation, MSPHL began prospective screening using the IDS assay on 1 November 2018. In the first 18 months of screening (to 30 June 2020), 146,954 specimens were prospectively screened using the method. Two newborns were identified with severe Hunter syndrome and the assay had a presumptive positive rate of 0.022%.

## 1. Introduction

Rates of newborn screening (NBS) for lysosomal storage disorders (LSDs) in the United States have soared following the addition of Pompe disease (Glycogen storage disease type II) and Mucopolysaccharidosis Type I (MPS I) to the Secretary of the Department of Health and Human Services’ Recommended Uniform Screening Panel (RUSP) in 2015 and 2016, respectively [1,2]. As new LSD treatments and new testing methods become available, it is anticipated that other LSDs will be added to the RUSP. Mucopolysaccharidosis Type II (MPS II), also known as Hunter syndrome, has been discussed for nomination to the RUSP [3]. MPS II is a rare X-linked lysosomal storage disorder caused by deficiency of the lysosomal enzyme iduronate-2-sulfatase (IDS) that affects an estimated 1 in 100,000–150,000 male births. As with other mucopolysaccharidoses, MPS II is clinically heterogeneous in terms of disease severity and onset. Due to the rarity of the disease and nonspecific symptoms, patients often undergo a lengthy clinical odyssey prior to diagnosis and the reported incidence of the disease is thought to be underestimated [4]. Clinical manifestations of Hunter syndrome include respiratory disease, cardiac disease, skeletal deformities, and intellectual disabilities. Affected boys often do not survive past the second decade of life due to respiratory or cardiac failure [5].

Two treatments, enzyme replacement therapy (ERT) and hematopoietic stem cell transplantation (HSCT), are the current standards of care for MPS II. The specific course of treatment for each patient is based on disease severity and presentation. ERT has been shown to be effective in treating somatic disease symptoms and can produce significant improvements in mobility and pulmonary function, especially when started during infancy [6,7]. HSCT has shown efficacy in treating central nervous system (CNS) symptoms, with improved benefit when the transplant is performed early in life [8,9]. For these reasons, identification of MPS II at birth is imperative for both severe and attenuated patients so that therapies can be initiated as soon as possible and the natural history of the disease can be modified [10,11].

The Missouri State Public Health Laboratory (MSPHL) has performed full-population NBS for five LSDs—Pompe disease, MPS I, Fabry disease, Gaucher disease, and Krabbe disease—for over seven years [12,13]. In July 2017, Missouri passed legislation adding Mucopolysaccharidosis Type II (MPS II) to their newborn screening panel. MSPHL validated a laboratory developed test (LDT) for MPS II, which is based on a fluorometric enzyme assay performed in a microwell plate format using FDA-registered analyte specific reagents (ASRs). In this report, we summarize the results of analytical validation studies, including linearity, sensitivity, precision, and carry-over. Preliminary cutoffs were determined during a clinical validation pre-pilot study using more than 5000 deidentified newborn dried blood spot (DBS) specimens and seven DBS specimens from patients confirmed with MPS II. Prospective newborn screening with this method began on 1 November 2018. By 30 June 2020, 146,954 samples had been screened and two newborns diagnosed with severe Hunter syndrome following positive first and second tier screens and positive confirmatory test results. These studies demonstrate a rapid, reliable method for high throughput NBS of MPS II that can be implemented using equipment that is readily available in most public health laboratories.

## 2. Methods

### 2.1. Dried Blood Spot (DBS) Specimens

For the analytical validation studies, quality control (QC) DBS specimens were obtained from Baebies, Inc. (Durham, NC, USA) and the Centers for Disease Control and Prevention (CDC) Newborn Screening Quality Assurance Program (Atlanta, GA, USA). Residual, deidentified DBS samples from more than 5000 NBS specimens obtained from Missouri newborns for routine screening were used for the clinical validation and pre-pilot enzymatic activity cutoff determinations. These specimens were stored at −20 °C to −30 °C in airtight bags with desiccant for approximately six years at the time of use. DBS from non-newborn patients affected with MPS II were obtained through Missouri’s contracted genetic referral centers. MSPHL received an IRB exemption for validation and screening for MPS II on 1 December 2017. During prospective screening, all samples received by the Missouri newborn screening program beginning on 1 November 2018 were tested using this method. Samples tested during prospective screening were stored at 2–8 °C after receipt and through the routine newborn screening procedure.

### 2.2. Reagents

Reagents and calibrants were purchased from Baebies, Inc. Ninety six-well flat-bottom black polypropylene half-area microtiter plates and clear adhesive plate sealers were obtained from Millipore Sigma (St. Louis, MO, USA). Ninety six-well clear round-bottom microtiter plates and adhesive aluminum plate-sealers were obtained from Fisher Scientific (Pittsburgh, PA, USA).

### 2.3. DBS Sample Extraction

To extract iduronate-2-sulfatase (IDS) enzyme from the DBS samples, one punch (3.2 mm) from each DBS was placed in individual wells of a clear, round-bottom 96-well microtiter plate. Sample extraction solution (100 μL; Baebies, Inc., Durham, NC, USA) was added to each sample well; the plate was covered with a clear adhesive sealer to prevent evaporation, and then incubated on a plate-shaker (600 rpm) at room temperature (RT) for 30 min.

### 2.4. Enzyme Activity Measurement

A detailed protocol for the IDS enzyme activity can be found in Supplemental document 1. Briefly, IDS activity was determined by adding 20 μL of the IDS substrate solution (Baebies, Inc.) to the appropriate wells of a black flat bottom, half-area 96-microwell plate. Ten microliters of extract from each sample was transferred to the black 96-well plate using a multi-channel pipette. The plate was then sealed tightly with an aluminum plate sealer and placed in an incubator at 37 °C for 2 h. Following incubation, the reaction was terminated by adding 50 μL of C-Stop reagent (Baebies, Inc.) to each reaction well. The plate was then re-sealed and mixed at 600 rpm on the plate shaker for 2 min at room temperature before centrifugation at 3700 rpm (2250× *g*) for 2 min. Lastly, 80 μL from the 4-methylumbelliferone (4MU) dilution set strip tube was added to the appropriate wells of the assay plate. The fluorescence of the plate was read in a BioTek Synergy HTX microtiter plate reader (Agilent; Santa Clara, CA) with 400 (±15) nm excitation and 485 (±20) nm emission filters; the fluorescence was measured as relative fluorescence units (RFUs). The RFUs obtained in the raw data output file from the plate reader were converted to activity units (μmol/L/h) using the following calculation (1):

Activity (μmol/L/h) = (RFU − NEH)/slope/incubation time/dilution factor(1)

where,

- RFU = Relative Fluorescence Units derived from the raw plate reader data output file
- NEH = Non-enzymatic hydrolysis (assay blank)
- Slope = slope of the linear calibration curve obtained from the RFUs and corresponding concentrations of the 4MU Dilution Set (Dil. A—D; 0.0375 μM − 0.3 μM)
- Incubation time = 2 h
- Dilution factor = 0.003875, calculated from the dilution of 10 μL of the DBS extract in a total of 80 μL of reaction volume, where the DBS extract is obtained from 3.1 μL of blood specimen from a single DBS punch in 100 μL of Extraction Solution (2):

(2) $$\mathrm{Dilution}\;\mathrm{factor}=\frac{3.1}{100}\times \frac{10}{80}=0.003875$$

### 2.5. Linearity

To test linearity, 10 quality control high (QCH) DBS punches and 10 quality control low (QCL) DBS punches were extracted and combined to make a QCH pool and a QCL pool. The two pools of extract were combined to generate a linear range of 10 sample dilutions of IDS enzyme activity (0%, 11%, 22%, 33%, 44%, 56%, 67%, 78%, 89%, and 100% of QCH activity). These dilutions were labeled sample 1 through sample 10, respectively. Four replicates from each sample dilution were organized randomly in the plate with the remainder of the wells filled by quality control low (QCL), quality control medium (QCM), and blank (extraction solution only) samples. The 4MU dilution set was loaded in the first column of the plate. The acceptance criteria were established based on the line of fit; an *R*^2^ value of >0.95 was required for acceptance.

### 2.6. Analytical Sensitivity

Limit of Blank (LoB) and Limit of Detection (LoD) were calculated by testing 80 replicates of a blank sample (extraction solution only), and 61 replicates of a specimen with low enzyme activity, quality control base pool (QCBP). The blanks and low activity specimens were loaded in columns 2–11 of the plate, with the 4MU dilution set loaded in column 1 and blank samples loaded in column 12. The mean and standard deviation of the blank samples (EXT) and the baseline samples (QCBP) were calculated and used in the equations below (3):

LoB = Mean_EXT_ + 1.645 (SD_EXT_) LoD = LoB + 1.645 (SD_QCBP_) (3)

### 2.7. Precision

Precision was analyzed by testing three replicate samples per plate of each of four levels of QC materials provided by the CDC (CDC-Base Pool, CDC-Low, CDC-Med, and CDC-High) over a total of five days. Three additional QC specimens from Baebies (QCL, QCM, and QCH) were also tested on each of the five days; the number of replicates per plate for each of the Baebies QCs was 7, 6, and 3, respectively. On each plate, the 4MU dilution set was loaded in column 1, 44 normal specimens (N) were loaded randomly, interspersed with quality control materials in columns 2–11, and column 12 was loaded with normal specimens and non-enzymatic hydrolysis assay controls. The data were compiled and analyzed for intra-run as well as inter-run precision with an allowable precision of ≤20% coefficient of variation (CV). The CV for each of the normal samples was also calculated to further evaluate precision.

### 2.8. Carry-Over

Ten punches of QCL DBS and eight punches of QCH DBS were extracted in individual wells of a microtiter plate, then pooled into one 1.5 mL microfuge tube for each level. The pooled extracts were loaded on two halves of a single assay plate with QCL in rows A–D, QCH in rows E–H, and four QCL samples placed among the QCH samples in rows F and G. The QCL samples placed in rows A–D were designated as “QCL-1” and the QCL samples placed among the QCH samples in rows F and G were designated as “QCL-2”. The enzymatic activity of QCL-1 was compared to the activity of QCL-2 to evaluate any carry-over from the QCH samples into the QCL-2 test wells.

### 2.9. Clinical Validation

Deidentified DBS specimens (*n* = 5301) were punched and extracted in 96-well plate format; each plate contained the appropriate calibrants, non-enzymatic controls, four QC specimens, and a single punch from 76 unique deidentified DBS specimens. While the specimens were deidentified before punching, general information including prematurity status (pre-term or full-term), age at collection (less than or greater than 24 h of age), and transfusion status (transfused or non-transfused) was retained with the sample to allow the effect of these variables to be analyzed. General non-parametric population statistics such as median, minimum, and maximum were calculated for each subgroup. Descriptive statistics such as mean and standard deviation could not be calculated for the patient population data without data transformation as the distribution shape is non-normal. Additionally, seven diagnostic samples from patients known to be affected with MPS II were also tested.

### 2.10. Prospective Screening

Prospective samples received by the Missouri newborn screening program were punched and extracted in the same plate layout described in Section 2.9. Based on the clinical validation, a two cutoff system was implemented; the retest cutoff was set at 40 μmol per liter per hour (μmol//L/h), and the high risk cutoff was set at 35 μmol/L/h. Samples with measured enzyme activity below the retest cutoff were repeated in duplicate; if the average activity value after retesting was below the high risk cutoff, the specimen was sent for second tier testing. On 2 August 2019, after approximately 10 months of prospective screening, the cutoffs were adjusted to 25 μmol/L/h (retest cutoff) and 20 μmol/L/h (high risk cutoff). From 1 November 2018 to 31 December 2019, second tier testing was completed at Greenwood Genetic Center (Greenwood, SC, USA) using molecular sequencing for all specimens with average activity below the high risk cutoff. From 1 January 2020 to the end of the study period, second tier testing was performed by Mayo Clinic Laboratories (Rochester, NY, USA) through evaluation of DBS glycosaminoglycan (GAG) concentrations. For samples with a positive second tier result from either Greenwood Genetic Center or Mayo Clinic Laboratories, the newborn was referred to the contracted follow up center for confirmatory testing. Additional biochemical testing was completed during follow up in these cases; this included urinary testing of GAG concentrations as well as other biochemical testing, family history, and physical examination. After follow-up testing, each case was classified using all available data.

## 3. Results

### 3.1. Linearity

**Figure 1:**
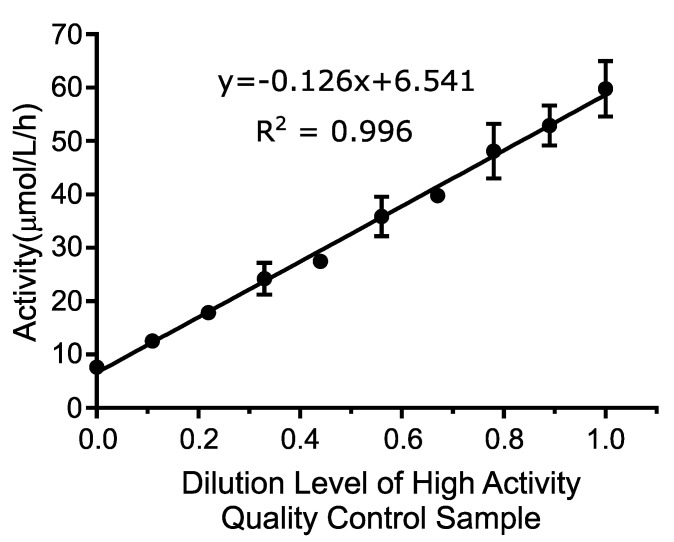
Linearity of the iduronate-2-sulfatase (IDS) rates of newborn screening (NBS) assay. Dilutions of the high QC samples were made in nine-step increments and plotted versus the activity detected. Four replicates were run at each dilution and the means +/− the standard deviation were graphed.

Linearity was determined by testing a range of blended DBS extract between 0% and 100% activity, where 100% activity is defined as the QCH sample. The average value of all four replicates of each sample level was calculated and plotted using the numeric sample level (1–10) on the *x*-axis and the mean activity value for each level (in μmol/L/h) on the *y*-axis. A line of best fit was assigned to the linearity results as shown in Figure 1. The *R*^2^ of the line was 0.996, which exceeded the acceptance criteria of >0.95.

The linearity was further evaluated by comparing the best linear fit to the best second and third order polynomial fit to calculate the non-linearity at each level. This analysis included all individual data points as opposed to average values using Analyse-It software, version 5.01. Neither the second order (*p*-value: 0.1483) or the third order (*p*-value: 0.8900) polynomial provided a better fit than the first order (linear) fit. These results indicate that the assay is linear between 7.6 and 59.7 μmol/L/h, which were the lowest and highest concentrations tested, and that the first order equation was the best fit over the measured range.

### 3.2. Analytical Sensitivity

Limit of Blank (LoB) and Limit of Detection (LoD) were determined by running 80 replicates of a blank sample (extraction solution only), and 61 replicates of a specimen with minimal enzyme activity (QCBP). The results in Table 1 show a LoD of 1.88 μmol/L/h and a LoB of 0.855 μmol/L/h. One blank result was determined to be a statistical outlier using Grubbs test (*z*-value: 6.94, critical value: 3.67) and was excluded from the analysis.

### 3.3. Precision

Within-run and overall precision were calculated for the six QC specimens tested (Table 2). Overall precision was calculated for each normal specimen, although within run precision could not be calculated for each normal sample as only a single replicate of each normal was tested on each plate. The assay met the reproducibility (overall precision) goal of 20% for all QC levels. Intra-run (single-day) precision ranged from 5.7–14.7% and multi-run precision ranged from 7.1–14.7% across the six QC samples. Overall precision was also calculated for each of the presumed normal samples tested. The median %CV of the normal samples was 8.0%. Precision of the CDC-BP level was not evaluated as it is formulated to have minimal functional enzyme activity and CV values are not statistically meaningful as enzyme activity approaches zero.

### 3.4. Carry-Over

Carry-over was assessed by placing the low activity quality control (QCL) samples within the plate layout immediately following a high activity quality control (QCH) sample. First, a series of QCL samples were run to obtain a baseline (QCL-1 samples). Secondly, high activity quality control samples were run with identical aliquots of QCL run interspersed amongst the QCH samples. These QCL samples are referred to as QCL-2 samples. If carry-over exists in this assay, it is expected that the values of the QCL-2 samples will be higher than those of the QCL-1 samples. If no carry-over exists, it is expected for the values to be roughly identical.

Results of carry-over testing are shown in Table 3. The mean value of all replicates for each QCL-1 and QCL-2 were calculated. The QCL-2 sample values (read adjacent to QCH) were not higher than QCL-1 samples that were separated from QCH samples. These results show that carry-over does not occur in this assay.

### 3.5. Clinical Validation

MSPHL tested 5301 deidentified (presumed normal) newborn DBS clinical samples to validate the clinical performance of the assay. This cohort was comprised of samples in the following groups: transfused (*n* = 153), premature (*n* = 152), collected prior to 24 h of life (*n* = 152), and normal (without another categorization; *n* = 4844). Premature samples were defined as those collected prior to 34 weeks of gestational age. Additionally, seven diagnostic samples collected from patients diagnosed with MPS II were also tested.

**Figure 2:**
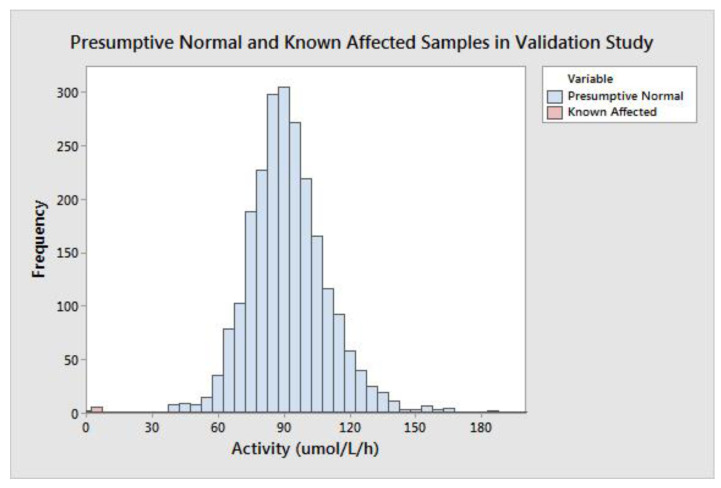
Population distribution of IDS activity (*x*-axis, reported as μmol/L/h) in 5301 presumed normal newborn dried blood spot (DBS) specimens (blue bars) and seven known affected Mucopolysaccharidosis type-2 MPS II patient DBS specimens (pink bars).

The group of all presumptive normal specimens is shown in Figure 2. The population median was 89.74 μmol/L/h, and the minimum value of a presumptive normal specimen was 29.38 μmol/L/h. MPS II is caused by a deficiency of IDS enzyme; very low activity results could be indicative of disease. The group of seven affected diagnostic samples ranged in activity from 1.90–7.00 μmol/L/h with a mean of 3.60 μmol/L/h and median of 3.15 μmol/L/h (Table 4). Of note, the six specimens with the highest activity values were not ERT naïve; that is, they had previously received enzyme replacement therapy, which can increase the measured enzyme concentration in the DBS.

The difference between the high-end of the affected range (determined by the affected sample with the highest activity) and the low-end of the presumed normal range (determined by the normal sample with the lowest activity) is 22.38 μmol/L/h. The spread between the affected and presumed normal populations can also be described in terms of measurement reproducibility; this gives a more accurate understanding of the potential for a false negative result due to assay variability. To calculate the spread in terms of standard deviation, the SD at the activity value of the highest affected sample (7.00 μmol/L/h) was calculated using linear interpolation of the precision results from the CDC-L and QCL samples. The calculated SD at 7.00 μmol/L/h was 0.89 (CV = 12.7%), meaning there are approximately 25 standard deviations between the highest affected and lowest presumed normal specimen. The large separation between the presumed normal and affected populations should allow for a minimal false positive rate with extremely low risk of false negative results.

### 3.6. Prospective Screening

**Figure 3:**
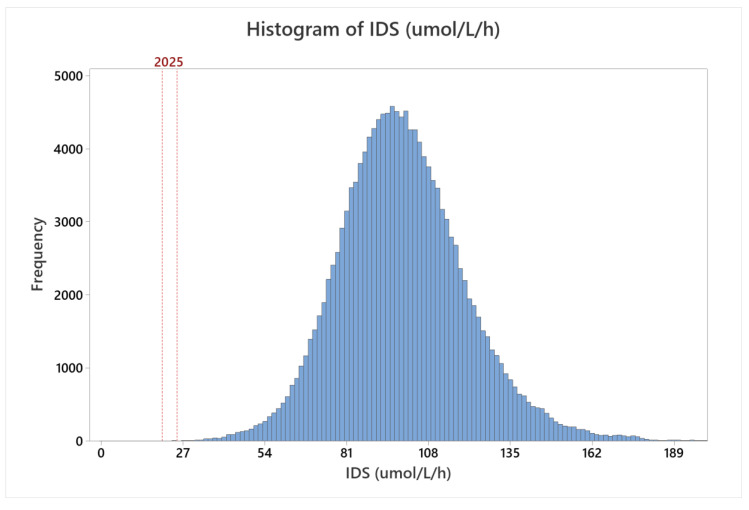
Distribution of IDS activity during prospective screening. The red dashed lines at 20 and 25 μmol/L/h represent the final high risk and retest cutoffs, respectively.

MSPHL began population screening for MPS II on 1 November 2018. From 1 November 2018 to 30 June 2020, 146,954 specimens were tested. The distribution of all specimens screened is presented in Figure 3. The shape of the population distribution was comparable to the shape of the distribution from clinical validation testing; however, the median activity was 98.29 μmol/L/h, which was approximately 10% higher than the median measured during clinical validation. The increase in activity relative to the clinical validation dataset could be due to a number of factors, including sample storage age or differences in sampling (for example, inclusion of a higher proportion of samples collected greater than seven days after birth, or seasonal fluctuations). 

During this period, 101 samples (0.07%) had activity below the retest threshold when first tested. Following retest, 44 samples (0.03%) had activity below the high risk threshold. Risk assessment was performed on these 44 specimens; 12 were determined to be low-risk based on results of a previous screen, abnormal results for a different NBS assay, or other factors. In the low-risk cases, a repeat specimen was requested. The remaining 29 samples were sent for second tier testing. The results of first tier enzyme testing (represented as the average of the initial test and retests of the same specimen), second tier testing, and any subsequent confirmatory test results are presented in Table 5. The assay screen positive rate (calculated as the number of specimens sent for second tier testing divided by the total number of specimens tested) was 0.022%.

Of the samples sent for second tier and confirmatory testing, two male newborns were determined to have severe Hunter syndrome. Case #13 in the table below began receiving enzyme replacement therapy (ERT) on 26 August 2019. This baby was diagnosed through amniocentesis and newborn screening, as the newborn had a positive maternal family history of MPS II. Immediately prior to the first ERT infusion, this newborn had elevated quantitative urine GAGs (161.3 mg/mmol; reference range ≤53 mg/mmol). Case #22 also began receiving ERT on 29 April 2020. This newborn also had a positive family history of MPS II.

Ten additional newborns were found to have variants of unknown significance in the IDS gene. Based on the confirmatory test results and the molecular variant analysis, these newborns are considered to have genotypes of unknown significance and will undergo long-term monitoring for emergence of symptoms. None of these newborns has begun ERT infusions to date. Three of the newborns were determined to have a pseudo-deficiency variant and nine others were classified as normal. Five additional cases are still pending follow up.

## 4. Discussion

Hunter syndrome, mucopolysaccharidosis type II (MPS II), is an X-linked lysosomal storage disorder characterized by a dysfunctional iduronate-2-sulfatase (IDS) enzyme. IDS functions in the pathway of heparan metabolism and a lack of IDS activity causes the accumulation of heparan sulfate and dermatan sulfate in several organs of the body [10]. Currently, MPS II is not a part of the RUSP, but some state governments in the United States have already begun mandating testing for the disease as a part of universal newborn screening. Conventional and HSCT are the primary therapy strategies for confirmed MPS II, but additional therapies such as intrathecal ERT, substrate reduction therapy, and gene therapy are under investigation to improve efficacy of treatment of central nervous system (CNS) dysfunction. The average age of symptom onset for MPS II has been reported as 1.5 years for severe MPS II and 4.3 years for attenuated MPS II. Both ERT and HSCT have shown reduced efficacy when initiated after presentation of ERT symptoms [11]. For these reasons, effective newborn screening assays such as the one described in this article are needed to identify affected individuals at birth.

This study represents the first reported pilot for MPS II newborn screening using a fluorometric enzyme assay method. Analytical validation of the assay revealed linearity (Figure 1), sensitivity (Table 1), precision (Table 2), and carry-over (Table 3) comparable to well established NBS assays. Retrospective screening of over 5301 presumed normal newborn DBS and seven DBS specimens from patients confirmed with MPS II revealed exemplary separation of the presumed normal and patient samples—greater than 25 standard deviations separating normal from affected specimens. Through prospective screening of more than 146,000 samples, the assay performance was excellent, resulting in a low screen positive rate (0.022%) while detecting two newborns with severe Hunter syndrome (estimated incidence rate during the study of 1 in 60,833 live births). Results of these studies demonstrate a rapid, reliable method for high throughput NBS of MPS II that can be implemented using equipment that is already available in most public health laboratories.

The clinical performance of this assay was also compared to published results from other newborn screening programs. Two other large newborn screening population studies of MPS II have been recently published; one study was conducted in Illinois [14] and the other in Taiwan [15]. Both studies utilized tandem mass spectrometry. The screen positive rates (0.009% and 0.050%, respectively) and estimated incidence rates (1 in 113,090 and 1 in 51,020, respectively) from these studies were comparable to the results described above.

This analysis showed that an assay for MPS II can be run in a time and cost-effective manner in a state public health laboratory. This test can also clearly discriminate between normal and affected samples using a highly precise and sensitive assay with little carryover or clinical overlap between the normal and affected populations. Given the availability of therapies that can alleviate disease systems, identifying MPS II in newborns is imperative to the early initiation of treatment and improved long term outcomes.

## Figures and Tables

**Table 1:** Sensitivity.

Blank Mean (μmol/L/h)	Blank SD (μmol/L/h)	LoB (μmol/L/h)	Low SD (μmol/L/h)	LoD (μmol/L/h)
0.075	0.505	0.855	0.62	1.88

**Table 2:** Precision.

Sample	Mean	Repeatability CV	Repeatability SD	Within Laboratory CV	Within Laboratory SD
CDC-L	6.050	11.9%	0.723	12.0%	0.725
CDC-M	25.558	7.4%	1.882	9.2%	2.353
CDC-H	48.028	5.7%	2.727	7.1%	3.421
QCL	9.610	14.7%	1.410	14.7%	1.410
QCM	29.140	11.2%	3.269	11.5%	3.356
QCH	56.909	8.2%	4.645	8.2%	4.645

**Table 3:** Assessment of Carry-over.

QCL-1 Mean (μmol/L/h)	QCL-2 Mean (μmol/L/h)	% Change
8.82	8.67	−1.70%

**Table 4:** Reported IDS Activity in MPS II Patient Samples.

Sample	IDS (μmol/L/h)	Age	Gender	Previously Received ERT?
LSD2001	2.06	21 months	M	No
LSD2002a	1.90	3 years	M	Yes
LSD2003a	3.37	2 years	M	Yes
LSD2004a	3.00	5 years	M	Yes
LSD2005a	3.15	7 years	M	Yes
LSD2006a	4.70	11 years	M	Yes
LSD2007	7.00	3 years	M	Yes

**Table 5:** First Tier, Second Tier and Confirmatory Test Results.

Case	Gender	IDS Activity (μmol/L/h)	Second-tier Results	Confirmatory Results	Confirmatory Diagnosis
1	M	31.6	normal banding pattern between intron 7 and exon 3 of the IDS-2 pseudogene was not observed	Normal GAGs	Normal
2	M	11.85	c.1205A > C (p.E402A)	Normal GAGs	Pseudo-deficiency
3	M	31.5	normal banding pattern between intron 7 and exon 3 of the IDS-2 pseudogene was not observed	Normal GAGs	Normal
4	M	11.73	Pseudo-deficiency c.684A > G & c.851C > T	N/A	Pseudo-deficiency
5	M	33.1	Normal	N/A	Normal
6	M	26.86	c.258G > C (p.P86P)	Normal GAGs	Hunter-Gen. of Unk. Sig.
7	M	29.54	normal banding pattern between intron 7 and exon 3 of the IDS-2 pseudogene was not observed	Normal GAGs	Normal
8	M	8.23	Hemizygous c.890G > A/p.R297H	Normal GAGs	Hunter-Gen. of Unk. Sig.
9	M	6.17	Hemizygous c.1417C > T/p.P473S	Normal GAGs	Hunter-Gen. of Unk. Sig.
10	M	17.63	Hemizygous c.754G > T/p.D252Y	Slightly abnormal heparan sulfate	Hunter-Gen. of Unk. Sig.
11	M	8.16	Hemizygous c.889C > G/p.R297G	Normal GAGs	Hunter-Gen. of Unk. Sig.
12	M	11.36	Hemizygous c.1499C > T/p.T500I	Normal GAGs	Hunter-Gen. of Unk. Sig.
13	M	2.53	Hemizygous c.1307delA/p.K436R * 4	Abnormal GAGs	Hunter-Severe
14	F	34.96	Heterozygous c.1499C > T/p.T500I	Normal GAGs	Hunter-Gen. of Unk. Sig.
15	M	13.77	Hemizygous c.1499C > T/p.T500I	Normal GAGs	Hunter-Gen. of Unk. Sig.
16	M	11.11	Pseudo-deficiency c.684A > G p.Pro228Pro Hemizygous& c.851C > T p.Pro284Leu Hemizygous	N/A	Pseudo-deficiency
17	M	9.28	Hemizygous c.1409C > T/p.S470L	Normal GAGs	Hunter-Gen. of Unk. Sig.
18	M	9.21	Hemizygous c.346T > G/p.F116V	Normal GAGs	Hunter-Gen. of Unk. Sig.
**Missouri 2nd tier method changed from sequencing to biochemical**					
19	F	8.61	Normal GAGs	N/A	Normal
20	F	18.78	Normal GAGs	N/A	Normal
21	F	15.02	Normal GAGs	N/A	Normal
22	M	2.27	Abnormal GAGs	c.1362_1365dupTGGT; p.N456Wfs * 2	Hunter-Severe
23	M	3.66	Abnormal GAGs	Pending	Pending
24	M	5.64	Slightly abnormal heparan sulfate	Pending	Pending
25	M	4.22	Normal	Pending	Pending
26	M	16.76	Normal	N/A	Normal
27	M	12.94	Normal	N/A	Normal
28	F	19.59	Slightly abnormal heparan sulfate	Pending	Pending
29	M	1.98	Normal	Pending	Pending

## Supplementary Materials

The following is available online at https://www.mdpi.com/2409-515X/6/4/79/s1, Supplemental document 1: Standard operating protocol for the IDS assay in the MSPHL.
