# Supplementary material for: Validation and Implementation of a Highly Sensitive and Efficient Newborn Screening Assay for Mucopolysaccharidosis Type II
Source: Int J Neonatal Screen. 2020 Oct 14;6(4):79. doi: 10.3390/ijns6040079 (PMC7711921; doi:10.3390/ijns6040079)
Supplement: Supplementary file 1 [file IJNS-06-00079-s001.pdf]

Supplemental Document: Standard Operating Protocol for the iduronate-2-sulfatase (IDS) Enzymatic Assay

1. SCOPE

This document pertains to the microplate-based, enzymatic assay for the quantitation of iduronate-2-sulfatase (IDS) in dried blood spots (DBS). This assay is considered a laboratory developed test (LDT) that is not FDA approved, and uses analyte specific reagents (ASRs), which are quality-regulated by the FDA.

2. USE

The intended use of the IDS enzymatic microplate assay is to screen newborns for an absence or deficiency of IDS enzymatic activity. The assay quantifies the IDS activity in DBS specimens from newborns. Specimens identified with deficient IDS activity are to be tested with a separate confirmatory, diagnostic method.

3. DEFINITIONS

IDS: Acronym for iduronate-2-sulfatase

4-MU: Acronym for 4-methylumbelliferone, a fluorimetric agent

DBS: Dried Blood Spot samples obtained from pricking the heel of a newborn between 24-48 hours after birth that is collected on specially manufactured absorbent filter paper. The blood is allowed to thoroughly saturate the filter paper and air-dried for several hours before being mailed to the Newborn Screening Lab.

QC DBS: A quality control dried blood spot consisting of pooled newborn umbilical cord blood at varying levels of enzyme activity. QC Base Pool (QCBP) has 0% of the enzymatic activity of normal pooled blood, QC Low (QCL) has 5% the activity, QC Medium (QCM) has 50% the activity and QC High (QCH) has 100% the activity. These blood spots are used as a control to test for the lower range of enzymatic activity (deficiency) and a normal activity (a negative patient).

NEH: Acronym for non-enzymatic hydrolysis sample, which is the reaction blank and consists of only extraction buffer, substrate reagent, and stop solution.

LSD: Lysosomal Storage Disorders are genetic conditions most often caused by a lack of an enzyme that eliminates intermediate metabolites from the body’s cells under normal conditions.

CV: Coefficient of Variation is a measure of the relative variation of distribution that is independent of the units of measurement, and is calculated by dividing the standard deviation by the mean of the data set, sometimes expressed as a percentage.

4. PROCESS & PROCEDURE

5.1. Materials

5.1.1. Chemicals/Solutions/Controls

Name	Supplier	Cat./Part Number
Extraction solution	Baebies	2114
C-Stop solution	Baebies	3673
IDS Substrate reagent	Baebies	3640
4-MU Dilution set, A-H set	Baebies	3952
QC-H DBS	Baebies	2275
QC-M DBS	Baebies	2274
QC-L DBS	Baebies	2273
QC-BP DBS	Baebies	2272
QC-H DBS (external ctrl)	Centers for Disease Control and Prevention (CDC)	
QC-M DBS (external ctrl)	CDC	
QC-L DBS (external ctrl)	CDC	
QC-BP DBS (external ctrl)	CDC	

5.1.2. Equipment

Equipment	Manufacturer	Model #
BioTek Synergy HTX Plate Reader	BioTek	S1LFA
Novus 8-channel pipettors (1-10 µL)	Thermo Fisher Scientific	46300000
Novus 8-channel pipettor (100-1200 µL)	Thermo Fisher Scientific	46300800
10-300 µL 8-channel VIAFLO pipette	Integra Biosciences	4623
Titer Plate Shaker	Corning	S2020-P4-COR
Plate centrifuge	Eppendorf	5804
Humidified Incubator (37°C)	Fisherbrand Isotemp	115-103-0513
Integra ASSIST Robot system	Integra Biosciences	4500

5.1.3. Software

- BioTek Gen5 IVD v2.06 (Winooski, VT)
- Microsoft Excel (Redmond, Washington)

5.1.4. Enzymatic assay procedure

The enzymatic assay is performed following the procedures outlined below.

5.1.4.1. Setup

- Obtain the reagents, DBS samples, and any materials necessary for the procedure.
- Allow the DBS specimen, including the QC Blood Spots to equilibrate to room temperature before use.

5.1.4.2. Sample extraction

- Obtain one 3.2 mm punch from each DBS specimen and each QC specimen, placing one punch per well in a new, disposable 96-well clear round bottom plate(s). Do not punch any DBS into the wells designated for NEH or CAL.
- Using the 100-1200 µL 8-channel pipette in stepper mode, add 100 µL of Extraction Solution to wells in columns 2-12 of each elution plate.
- Seal the extraction plate(s) with clear adhesive plate sealer(s) using a roller. Place the sealed extraction plate(s) on the plate shaker.
- Shake the extraction plate(s) at 600 rpm for 30 minutes at room temperature. Use a calibrated timer set to 30 mins to track the extraction time.
- Obtain the appropriate number of IDS Reagent vials needed to run the samples (each vial is sufficient for 176 samples, including QCs and blanks, or two assay plates).
- Place the IDS Reagent into a water bath at room temperature, ensuring the lid of the reagent is above the water level. Let the reagent thaw for 10 minutes. Remove from the water bath and wipe dry with a lint-free cloth.
- After the 30 minute sample extraction is complete, remove and discard the clear adhesive plate sealer(s) from the extraction plate(s).

5.1.4.3. IDS enzymatic reaction

- Inspect the IDS Reagent and ensure that it is completely thawed. Vortex the thawed reagent vigorously for 30 seconds.
- Pour the IDS Reagent into a 10 mL reservoir on the Integra ASSIST robot's staging area.
- Label a 96-well half area black flat bottom assay plate with the plate ID and place on the plate holder on the Integra ASSIST robot. With the 300 µL Integra VIAFLO pipette attached and connected via Bluetooth, run protocol "20µL" to dispense 20 µL of IDS Reagent to columns 2-12 of the assay plate.
- Remove the assay plate from the Integra ASSIST and using the 1-10 µL 8-channel pipette, transfer 10 µL of each sample extract to the

appropriate reaction wells, ensuring that the tips touch the bottom of the well. Use new tips for each column of samples to avoid contamination between wells.

- Tightly seal the assay plate(s) with aluminum adhesive plate sealer(s), using a roller to ensure a tight seal.
- Place the sealed assay plate(s) in an incubator set at 37°C. Do not stack assay plates on top of each other.
- Incubate for 2 hours ±5 minutes, using a calibrated timer set for 2 hours to ensure accurate incubation time.

5.1.4.4. Stop reactions and read plate(s)

- Ten minutes prior to the end of the 37°C incubation, obtain the 4-MU Dilution Set from the freezer and C-Stop bottle stored at room temperature. One 8-tube strip of the 4-MU Dilution Set and one bottle of C-Stop can be used for up to two assay plates.
- Allow the 4-MU Dilution Set to thaw at room temperature for approximately 5 minutes in a room temperature water bath, ensuring that the top of the tubes are above the level of the water.
- Remove the assay plate(s) from the incubator upon completion of the 2 hour incubation.
- Peel back the aluminum adhesive plate sealer(s) to expose all of the reaction wells while leaving the sealer attached to the right edge (near column 12) of the assay plate.
- Fill the 10 mL reagent reservoir with C-Stop Solution and use the 300 µL Pipette with the “50µL” protocol to add C-Stop Solution to wells in columns 2 – 12 of each plate.
- Remove the completed plate from the ASSIST and place the next assay plate on the plate holder. Press “RUN” on the pipette to resume addition of C-Stop Solution to the second plate.
- Refill the reservoir with more C-Stop Solution as needed, being careful not to spill any liquid out of the reservoir. Repeat the “50µL” dispense protocol for each set of two assay plates.
- Re-apply the aluminum plate sealers to the plate(s) and mix the assay plate(s) at 600 RPM on the plate shaker for 2 minutes (using a timer) at room temperature.
- Spin the assay plates in the plate centrifuge with microplate adaptor at 3,700 rpm (2,250xg) for 2 minutes. NOTE: Properly balance the plate centrifuge. If the run contains an odd number of assay plates, balance with an empty plate. After the spin, carefully remove and discard the aluminum plate sealers.
- Using the 30-300 µL 8-channel pipette in stepper mode, add 80 µL from the 4-MU Dilution Set strip tube to the wells in column 1 of each assay plate (refer to Figure 1 for plate layout).
- Read plates on the microtiter plate reader in the Gen5 software.

5.1.4.5. Activity calculation

- The Relative Fluorescence Units (RFUs) obtained in the raw data output file from the plate reader are converted to activity units (µmol/L/h) using the following calculation:

Activity (µmol/L/h) = (RFU – NEH)/slope of 4-MU linear curve/incubation time/dilution factor

Where,

RFU = Relative Fluorescence Units derived from the raw plate reader data output file
NEH = Non-enzymatic hydrolysis (assay blank)
Slope of 4-MU linear curve = slope of the linear calibration curve obtained from the RFUs and corresponding concentrations of the 4-MU Dilution Set (Dil. A – D; 0.0375 µM – 0.3 µM)
Incubation time = 2 hours
Dilution factor = 0.003875, calculated from the dilution of 10 µL of the DBS extract in a total 80 µL of reaction volume, where the DBS extract is obtained from 3.1 µL of blood specimen from a single DBS punch in 100 µL of Extraction Solution, i.e.,

$$Dilution\ factor = \frac{3.1}{100} x \frac{10}{80} = 0.003875$$
